# Utilizing ANN for Predicting the Cauchy Stress and Lateral Stretch of Random Elastomeric Foams under Uniaxial Loading

**DOI:** 10.3390/ma16093474

**Published:** 2023-04-29

**Authors:** Zhentao Liu, Chaoyang Wang, Zhenyu Lai, Zikang Guo, Liang Chen, Kai Zhang, Yong Yi

**Affiliations:** 1 School of Materials and Chemistry, Southwest University of Science and Technology, Mianyang 621010, China; liuzhentao022@163.com (Z.L.);; 2 Research Center of Laser Fusion, China Academy of Engineering Physics, Mianyang 621900, China

**Keywords:** elastomeric foam, constitutive model, ANN, porosity, cell size

## Abstract

As a result of their cell structures, elastomeric foams exhibit high compressibility and are frequently used as buffer cushions in energy absorption. Foam pads between two surfaces typically withstand uniaxial loads. In this paper, we considered the effects of porosity and cell size on the mechanical behavior of random elastomeric foams, and proposed a constitutive model based on an artificial neural network (ANN). Uniform cell size distribution was used to represent monodisperse foam. The constitutive relationship between Cauchy stress and the four input variables of axial stretch λ_U_, lateral stretch λ_L_, porosity φ, and cell size θ was given by con-ANN. The mechanical responses of 500 different foam structures (20% < φ < 60%, 0.1 mm < θ < 0.5 mm) under compression and tension loads (0.4 < λ_U_ < 3) were simulated, and a dataset containing 100,000 samples was constructed. We also introduced a pre-ANN to predict lateral stretch to address the issue of missing lateral strain data in practical applications. By combining physical experience, we chose appropriate input forms and activation functions to improve ANN’s extrapolation capability. The results showed that pre-ANN and con-ANN could provide reasonable predictions for λ_U_ outside the dataset. We can obtain accurate lateral stretch and axial stress predictions from two ANNs. The porosity affects the stress and λ_L_, while the cell size only affects the stress during foam compression.

## 1. Introduction

Elastomeric foams exhibit nonlinear elasticity, fatigue resistance, and high flexibility due to their matrix material and cell structure characteristics. Various foams prepared through physical or chemical foaming are widely used in daily life and engineering [1,2]. They can insulate devices from external heat, humidity, and chemical environments. The random cells make foams lightweight and compressible, so they can recover to their original shape after suffering large deformation. Because of this typical mechanical behavior, elastomeric foams are commonly employed as cushioning materials [3]. Foam pads between two rigid surfaces typically withstand uniaxial loads [4,5]. It is known that the mechanical behavior of cellular solids is directly related to the cell wall material, the cell geometry, and the porosity [6]. However, owing to the random open or closed cell structures, the deformation process of elastomeric foam is very complex [7]. In order to accurately apply them to different industrial fields, a constitutive model that can describe the stress–strain relationship of foams under uniaxial loading is essential.

The current mainstream hyperelastic models are mainly based on strain energy functions to describe the mechanical behavior of elastomeric foams [8,9,10,11,12]. This is a polynomial form consisting three strain invariants or stretch ratios with material constants. The stress tensor can be obtained by taking the strain derivative of the function. We can obtain the constitutive model parameters through the following two steps: performing mechanical tests of the sample and fitting the experimental stress–strain curves with the strain energy function. However, since one parameter combination cannot cover the mechanical behavior of different porosities or cell structures, we have to repeat the above steps when preparing another new foam sample. Several studies have considered the effects of porosity on strain energy function [13,14,15], but they still require stress–strain data and do not contain the cell structure information. Some experiments also studied the relationship between cell microstructure and mechanical properties of elastomeric foams, but large porosity and cell size often occur together [16,17]. Therefore, in order to make up for the deficiencies of traditional homogenized foam constitutive models, it is necessary to establish a constitutive model that takes into account porosity and cell size.

Recently, machine learning (ML) and deep learning (DL) methods have shown great potential in material design and discovery due to their powerful predictive capabilities [18,19]. In mechanics, by training on experimental or simulation data, ML and DL models can quickly give mechanical properties based on material structure [20]. For example, Bessa et al. designed the structure of metamaterials into supercompressible ones with the guidance of Bayesian machine learning [21]; they used Gaussian process (GP) to predict the mechanical response for different structure parameters. Zheng et al. also studied the inverse relationship between the structure and mechanical properties of cellular materials using DL models (GAN) [22,23]. ML models have also been used as constitutive equations to describe the nonlinear mechanical behavior of materials in researches [24]. Artificial neural networks (ANNs) perform better than tree-based models in such continuous value prediction tasks due to their flexibility and approximation capabilities. Shen et al. and Liang et al. constructed strain energy forms for rubber and elastomeric foam materials with neural networks [25,26]. Jang et al. used an ANN to predict elastoplastic behavior for J2-plasticity [27]. The constitutive artificial neural networks (CANNs) proposed by Lin et al., which combine the theoretical knowledge of materials [28], can predict nonlinear or anisotropic behavior without stress–strain data. Ari Frankel et al. simulated the response of five porosity BCC closed-cell elastomeric foams under complex deformations and introduced a discrepancy method to improve the extrapolation ability of ML models [29]. In addition to providing accurate curve fittings, increasing input variables allows ML models to contain more structure parameters than traditional constitutive models.

**Figure 1:**
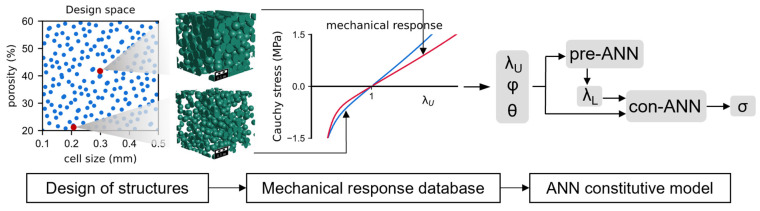
Data-driven process of constructing the ANN constitutive model.

This article focuses on random spherical closed-cell elastomeric foams and their mechanical behavior under uniaxial loading. To address the limitations of traditional phenomenological constitutive models, we propose an ANN constitutive model, which considers the effects of porosity and cell size. The cell size distribution is set to uniform. The data-driven process is shown in Figure 1 and as follows: (1) determine the boundary of the design space, sample the input variables, (2) perform mechanical simulations for foams with different structures, generate training data, (3) employ artificial neural networks to construct a constitutive model with the simulation data, and predict the mechanical response for various structures. In the following sections, we first present the specific form of the ANN constitutive model and then introduce the modeling and simulation process for elastomeric foams. We combine two ANNs to provide predictions for lateral stretch and Cauchy stress. Finally, we use physical empirical knowledge to improve the interpretability of neural networks beyond training data. We also compared ANN with Polynomial regression and Gaussian process regression models.

## 2. Materials and Methods

### 2.1. ANN Constitutive Equation

The ANN constitutive model used Cauchy stress instead of strain energy as the target value for the regression task. This is because when the deformation rises, the stress will increase rapidly, resulting in sparse training data and increased strain energy error calculated by numerical integration. Thus, the ANN constitutive equation pursues Cauchy elasticity rather than hyperelasticity. The stress tensor in a Cauchy-elastic material is defined as a function of the deformation gradient ***F***, which can be given by three principal stretches $\sigma =\bar{\sigma }\left(\lambda_{1},\lambda_{2},\lambda_{3}\right)$. In the case of uniaxial loading, λ_1_ = λ_U_, λ_2_ = λ_3_ = λ_L_, where λ_U_ and λ_L_ represent axial and lateral stretches, respectively. Hence, in our work, the constitutive equation for elastomeric foams taking into account porosity and cell size is

(1) $$\sigma_{U}=f(\lambda_{U},\lambda_{L},\varphi ,\theta )$$

where φ is the porosity of foam at the undeformed state, and θ indicates the cell diameter; ANN gives the nonlinear mapping between axial Cauchy stress and four input variables. In engineering applications, the φ and λ_U_ are given by

(2) $$\varphi =1-\mathrm{RD},\lambda_{U}=1+\varepsilon_{\mathrm{Eng}},$$

RD is the relative density of the foam; $\varepsilon_{\mathrm{Eng}}$ is the engineering strain on the loading direction and is negative when the foam is under compression.

As we used lateral traction-free boundary conditions in the simulation, λ_L_ is calculated by the average lateral strains rather than a fixed Poisson’s ratio. However, obtaining the lateral displacement from a uniaxial mechanical test requires additional equipment; thus, researchers may only have data on (λ_U_, φ, θ) in the usual scenario. For this problem, we proposed another pre-ANN to give λ_L_ and describe it in Section 2.4.

### 2.2. Design Space Definition

Firstly, it is necessary to determine the upper and lower bounds of input variables in the design space. The ranges of porosity, cell size, and axial stretch are shown in Table 1 and defined as follows: (1) Determine the structure domain and cell size. Studies have shown that the cell size of elastomeric foams is typically between one hundred microns and one millimeter, and the larger the cell, the less homogeneous the foam [1,30]. Since we used uniform cell size distribution to represent monodisperse foam [31], a structure size of 2 × 2 × 2 mm^3^ and a voxel length of 10 μm were chosen to allow the model to include the largest cell possible while saving computation time and resources. Then, the cell size range was set to 100~500 μm. (2) Determine the porosity range. Because we only allow tiny overlaps for spherical cells (1%), random foams cannot reach the theoretical maximum porosity of 64% (packing limit for monodisperse spheres), and foams with low-density have more anisotropy [32,33]. This is because low-density foams exhibit a wide variation in cell size and shape during preparation [34]. On the other hand, the location of the cells is random, and if a small porosity is set, the foam domains with large cell size may have density inhomogeneity. To make the foam domain isotropic and representative, we set the porosity bound to 20~60%. (3) Determine the axial stretch. The compression process for an elastomeric foam can be divided into three stages (linear, cell walls bending, and densification) [26]. We chose a moderate compressive strain to cover the region of cell walls bending. The thickness of the cell walls varies in a large range due to the random cells. We found that the stress–strain curve of the foam showed a cliff drop at a large tensile strain (λ_U_ > 3). This indicates that the thinner cell walls may experience fracture. For the above reasons, we set the λ_U_ = 0.4 and λ_U_ = 3 as the endpoints of the compression and tension loading paths for every foam model.

### 2.3. Numerical Simulation

**Figure 2:**
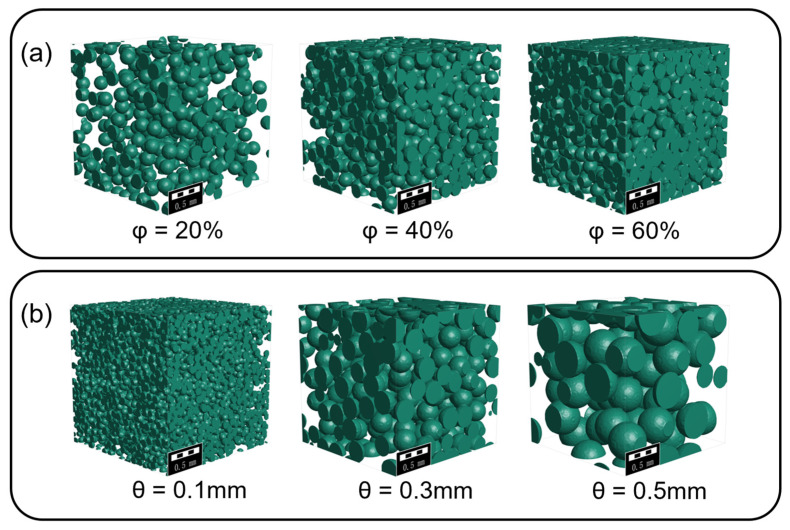
Grain models with different porosity and cell size; random foams are generated by inverting solid and gas phase materials. (**a**) θ = 0.2 mm, (**b**) φ = 40%.

Geodict software (Ver. 2017, Math2Market GmbH, Kaiserslautern, Rheinland-Pfalz, Germany) [35] accomplished the structure modeling and large deformation simulations of random elastomeric foams. It simulates porous media using finite volume and FFT-based methods [36]. Five hundred foam structure designs are generated by sampling the (φ, θ) space based on a Sobol sequence [37]. Next, we created the random elastomeric foams in two steps with the GrainGeo module, which is usually used to generate granular structures. First, we regarded the cells as solid particles, created a random grain model according to a combination of porosity and cell size in the Sobol sequence, and then inverted their material to air. Figure 2 shows the grain models created in the first step; the spherical solid phase will be inverted to void space to generate random foams. We used the Creat Grains part to define the foam structure, including domain size, voxel length, cell overlap, porosity, cell size, cell shape, and cell size distribution.

Uniaxial compression and tensile simulations were performed for all foam structures using the ElastoDict module. We chose silicone rubber with a neo-Hookean hyperelastic model as the matrix material [38]. When considering slight compressibility, the strain-energy function can be written as

(3) $$U=C_{1}\left({\bar{I}}_{1}-3\right)+\frac{1}{D_{1}}{\left(J-1\right)}^{2}$$

where ${\bar{I}}_{1}$ is the first invariant of isochoric left Cauchy Green deformation tensor $\bar{B}$, J=det(F), is a measure of the volume change. C_1_ and D_1_ are material constants which can be defined by the initial shear modulus G and bulk modulus K. To be more intuitive, we used an initial Young’s modulus E=2.7MPa [39] and a Poisson’s ratio ν=0.499 to derive the values of C_1_, D_1_:

(4) $$C_{1}=\frac{G}{2}=\frac{E}{4(1+\nu )},D_{1}=\frac{2}{K}=\frac{6(1-2\nu )}{E}$$

FeelMath-LD solver was used to conduct the large deformation simulation. Lateral traction-free and periodic boundary conditions were applied to both compression and tension simulations. A total of 1000 simulations were performed on 500 structures. We took 100 strain points on the loading path and obtained their corresponding average Cauchy stresses and lateral strains. The data-driven framework developed by Python and Geodict was used to automate the above modeling and simulation tasks. Structure parameters (porosity, cell size) and deformation states (λ_U_, λ_L_) with their mechanical response (Cauchy stress) are stored in a large dataset (100,000 samples). Next, using these data, two ANNs analyzed the constitutive behavior of elastomeric foams.

### 2.4. ANN Model

After generating the training data, a regression task was required to find the relationship between the four inputs and one output values. Given that this is a nonlinear function, we chose ANN to construct the constitutive equation. We used two feedforward neural networks, pre-ANN and con-ANN, to establish the constitutive model for random elastomeric foams under uniaxial loading.

First, we used pre-ANN to solve the problem described in Section 2.1: the lack of lateral stretch data in practical applications. We know a prior that the λ_L_ is closely related to λ_U_ and may also be related to the material’s microstructure. Therefore, we considered λ_L_ as a function of λ_U_, φ, and θ, and used pre-ANN to establish the relationship.

After completing pre-ANN training, we fixed the network and started training con-ANN. At this point, λ_L_ became an intermediate variable of two neural networks and served as the input of con-ANN. The weights and biases of con-ANN determine the specific form of the constitutive Equation (1). By combining the two neural networks, we can obtain the axial Cauchy stress using only three input variables. Thus, (1) is modified to

(5) $$\sigma_{U}=g(\lambda_{U},\varphi ,\theta )$$

The biggest problem faced by neural networks is to reduce the generalization error, including interpolation and extrapolation [40]. Neural Networks typically can make accurate and reasonable predictions for interpolated data but not for extrapolated data. We used physical empirical knowledge to improve the interpretability of two neural networks here:

(1) Choosing the appropriate input variable form: According to the equation $J=\det(F)=\lambda_{1}\lambda_{2}\lambda_{3}=\lambda_{U}\lambda_{L}{}^{2}$ in our case, there is an inverse relation between the two stretches. It means that the λ_L_ should approach infinity when λ_U_ closes to zero. Therefore, we replaced λ_U_ with 1/λ_U_ as the input of pre-ANN.
(2) Choosing the appropriate activation function: Theoretically, stress will become infinitely large as deformation continues. Common activation functions such as *tanh* and *sigmoid* are bounded functions; although they can provide good nonlinear fitting within the dataset, they cannot be extrapolated to cases where λ_U_ is very large. We chose the *softplus* activation function (6) for our ANNs to meet this requirement. The output of the *softplus* monotonically increases with the increase of the input.
  (6) softplus(x)=ln(exp(x)+1)

**Figure 3:**
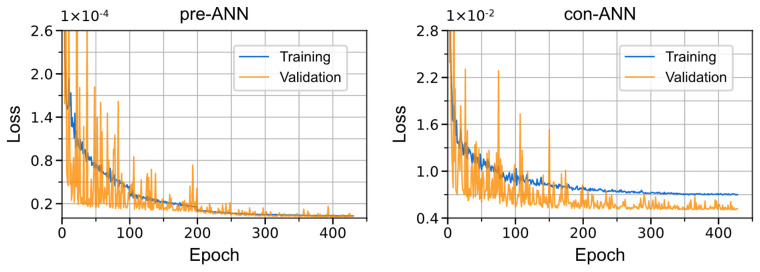
Training curve of two ANNs.

The pre-ANN had two fully connected hidden layers with eight nodes per layer, and the con-ANN had four hidden layers with 32 nodes per layer. Both networks used the *softplus* activation function in the hidden layer nodes, and the linear activation function in the output node. The mean squared error (MSE) was used as the loss function for two ANNs. We chose the Adam optimizer, and used EarlyStopping and ReduceLROnPlateau callbacks to prevent overfitting and update the learning rate. EarlyStopping callback would terminate the training when the validation loss was no longer decreasing for 60 epochs; ReduceLROnPlateau callback would reduce the learning rate by a factor of 0.8 when the validation loss stopped improving for 25 epochs. An amount of 20% of the whole dataset was split as test data, and 20% of the training data was used as validation data. The training processes are shown in Figure 3, and we used the TensorFlow-based Keras library to build our models.

## 3. Results and Discussion

### 3.1. Model Evaluation

**Figure 4:**
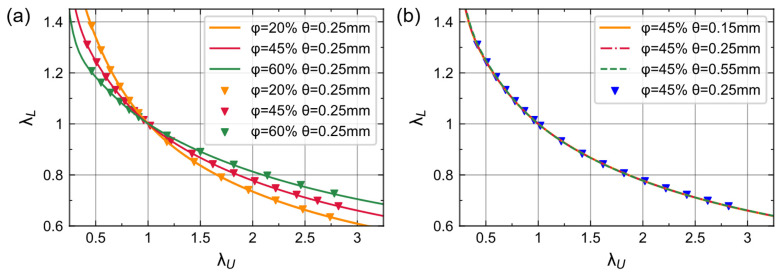
Lateral stretch of different structures predicted by pre-ANN; scatters are corresponding simulation data. (**a**) θ = 0.25 mm. (**b**) φ = 45%.

**Figure 5:**
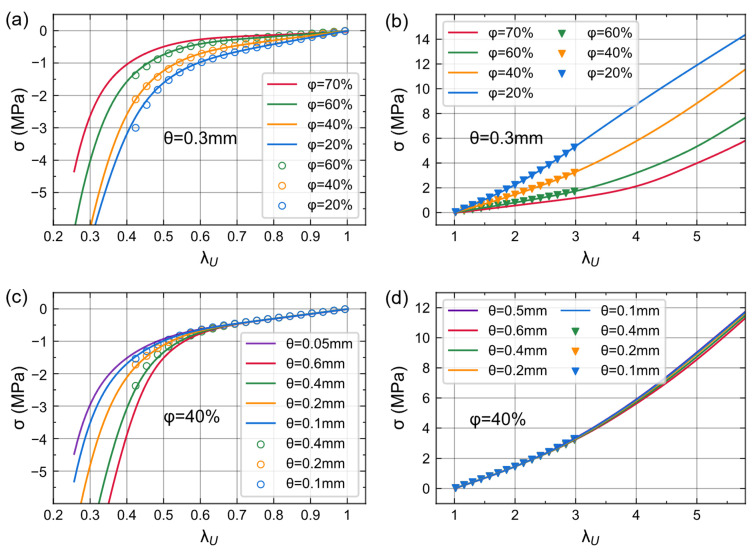
Stress-stretch curve of different structures predicted by con-ANN. Scatters are simulation data, red and purple lines represent porosity or cell size outside the dataset. (**a**,**b**) θ = 0.3 mm. (**c**,**d**) φ = 40%.

Neural networks usually perform well on the training set, but there is a risk of overfitting, which may lead to significant prediction errors on unknown data. After training, we evaluated the performance of two neural networks on the test data. R^2^ (coefficient of determination) and MAPE (mean absolute percentage error) were used as metrics for both regression models. R^2^ represented the fitting degree of the predictions, while MAPE measured the relative error of the two values. The scores of the two ANN models on test data are shown in Table 2. The MAPE of pre-ANN is extremely small, and the R^2^ is near to one, which indicates that the neural network’s prediction is very close to the actual value λ_L_ for each structure with three input variables. It prevents the λ_L_ that input to the con-ANN from accumulating too much error. Figure 3 shows that the training stops quickly once the loss becomes stable, which prevents overfitting. The larger learning rate (1 × 10^−3^) at the beginning of training makes the validation curve oscillate. The training set has more samples, so the average loss calculated on all batches is smoother. A small initial learning rate (1 × 10^−4^) would reduce the oscillation, but created local minima. The λ_L_ and Cauchy stress of different simulations predicted by the two ANNs can be seen in Figure 4 and Figure 5.

### 3.2. Predictions of λ_L_ and Cauchy Stress

We used scatter plots to represent the data calculated by Geodict and lines to represent neural network predictions. As shown in Figure 4, the pre-ANN can give accurate and smooth predictions of λ_L_ for different foam structures. Foams with high porosity have relatively small changes in λ_L_ during both deformation processes (Figure 4a). In contrast, cell size does not contribute to the value of λ_L_ (Figure 4b). Therefore, we can infer that the lateral stretch only relates to the axial stretch and porosity.

Figure 5a,b shows the uniaxial Cauchy stress of foams with different porosities. The cell size was set to 0.3 mm. The Cauchy stresses of three foams with increasing porosity (20%, 40%, 60%) were simulated by Geodict and predicted by con-ANN. We also utilized con-ANN to predict a structure that was not present in the dataset (porosity 70%). The results demonstrated that con-ANN’s predictions could fit the simulation data well and were consistent with prior physical knowledge. The corresponding stress gradually decreased with an increase in foam porosity under identical deformation conditions. In addition, con-ANN exhibited a certain degree of extrapolation interpretability. During foam compression, the linear stage and the cell walls bending stage can be regarded as one stage.

Then, we performed the same analysis on foams with different cell sizes (Figure 5c,d). The porosity was set to 40%. The analysis considered three cell sizes in the dataset (0.1 mm, 0.2 mm, 0.4 mm) and two outside the dataset (0.05 mm, 0.6 mm). During the initial stage of compression, there is little difference in stress. As deformation progresses, larger cells exhibit higher compressive stresses. This indicates that cell size can affect the compression stress of the foam. In the tension case, the stresses of the five foams with different cell sizes show little variation across the entire strain range. The larger cell shows a slightly smaller stress. Rostami-Tapeh-Esmaeil et al. reported that the tensile properties of elastomeric foams are more related to porosity, while the compressive properties depend on both porosity and cell size [41]. Heydari et al. indicated that larger cell sizes lead to more significant structural defects and more stress concentration [42]. The Cauchy stresses predicted by our con-ANN show the same trends.

### 3.3. Extrapolations of λ_U_

To provide interpretable lateral stretch and Cauchy stress predictions for λ_U_ outside the dataset, we used 1/λ_U_ as the input for pre-ANN and *softplus* as the activation function for two ANNs. When λ_U_ is close to zero in a compression case, the input of pre-ANN itself becomes infinite, so the neural network can provide a larger forecast without employing large weights. The situation is also the same in the tensile state. This improves the stability and rationality of pre-ANN.

**Figure 6:**
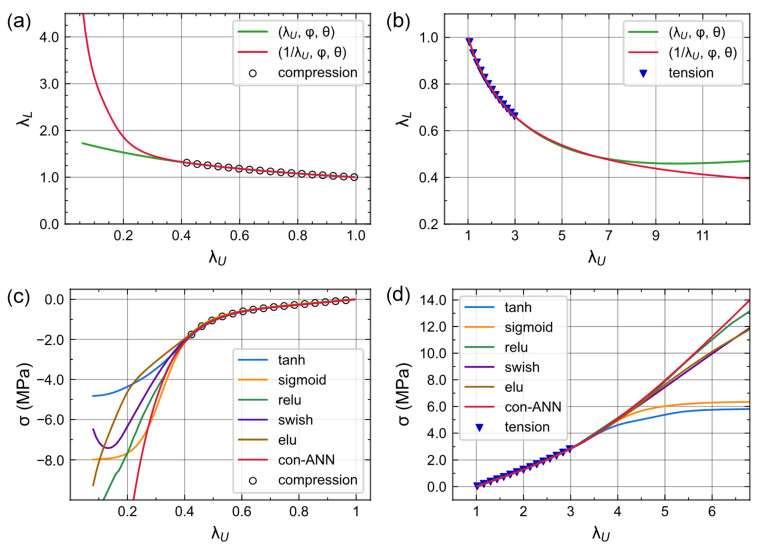
Extrapolation performance of two ANNs. The scatters are simulation data for a foam φ = 45%, θ = 0.25 mm. (**a**,**b**) Extrapolation of pre-ANN with different input forms. (**c**,**d**) Extrapolation of con-ANN with different activation functions.

Figure 6a,b shows the extrapolation performance of neural networks with different input forms. We can see that the pre-ANN’s predictions for λ_U_ outside the dataset are more physically reasonable. In compression, λ_L_ increases to a considerable value as λ_U_ decreases gradually to zero (Figure 6a). Additionally, λ_L_ decreases monotonically when the foam is stretched (Figure 6b). In contrast, a neural network that uses λ_U_ as the input cannot predict this inverse relationship between the two stretches when extrapolating, even though it can fit well within the dataset.

Unlike the *tanh* and *sigmoid* functions, the *softplus* function was unconstrained in its output and could accommodate the monotonic increasing relationship between λ_U_ and stress. The function curve is similar to the commonly used linear activation function, *ReLU*. We compared the extrapolation of neural networks using six different activation functions (*tanh*, *sigmoid*, *relu*, *swish*, *elu*, *softplus*). As shown in Figure 6c,d, all six activation functions successfully fit the simulation data, but only *softplus*, *ReLU*, and *elu* can reflect the monotonic relationship between stress and strain. The predicted stress values using *tanh* and *sigmoid* activation functions tended to stabilize for λ_U_ outside the data set. The *swish* curve even had an inflection point in compression case. Meanwhile, the linear activation function *ReLU* could not provide a relatively smooth prediction. The *elu* function also had a linear region(0.2 < λ_U_ < 0.4). Finally, the con-ANN equipped with *softplus* can provide reasonable stress predictions for strain in the entire deformation range of foams. It proves that the *softplus* function is suitable for such nonlinear regression tasks.

### 3.4. Comparison with Polynomial and GPR

We compared the performance of con-ANN, Polynomial regression and Gaussian process regression. All three models can be used for the nonlinear regression task in this paper. Polynomial regression used the polynomial of four input variables to model the Cauchy stress. We improved the model’s fitting ability by increasing the order of the polynomial (*n* = 2, 3, 4, 5). Gaussian process regression is a nonparametric, Bayesian machine learning model for nonlinear function [43]. It can provide a reliable estimate of their uncertainty. However, GP regression has O(N^3^) time complexity and O(N^2^) memory complexity; N is the number of training samples. In our 100,000 samples dataset, the covariance matrix of a full GP regression can be very large, leading to a sharp increase in computational cost. To address this limitation, we randomly sampled 3% of the training set and conducted a sparse GP regression [44].

**Figure 7:**
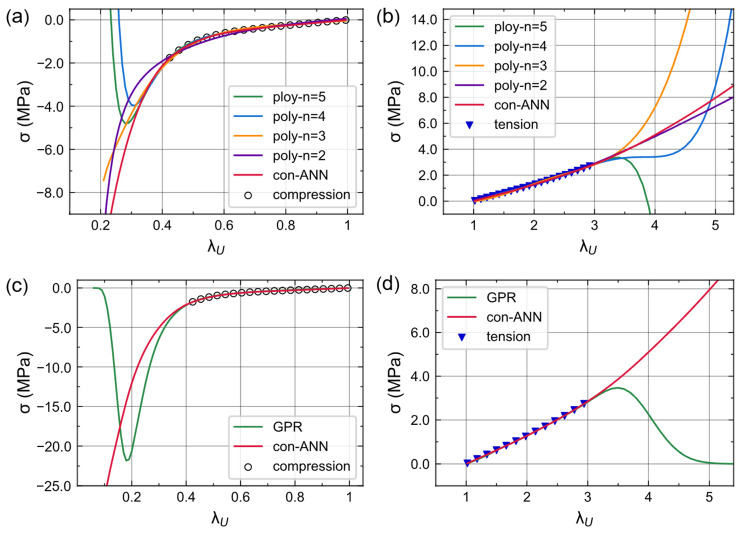
Extrapolation performance of con-ANN, polynomial regression, and GP regression. Scatters are simulation data for a foam, φ = 45%, θ = 0.25 mm. (**a**,**b**) Comparison of con-ANN and polynomial regression. (**c**,**d**) Comparison of con-ANN and GP regression.

As shown in Table 2 and Figure 7, a low-order polynomial (*n* = 2, 3) cannot fit the data well, while high-order polynomial models (*n* = 4, 5) are less reliable than con-ANN when extrapolating (Figure 7a,b). On the other hand, GPR fits very well within the dataset, but it tends to give a zero prediction when λ_U_ is outside the dataset (Figure 7c,d). This is due to the a priori assumption that the stress follows a Gaussian distribution with a mean of zero. Based on Table 2 and Figure 7, con-ANN has a smaller prediction error and interpretable extrapolation capability than the other two models.

### 3.5. Applications

Since pre-ANN can predict accurate lateral stretch, we can use it to obtain the dynamic Poisson’s ratio during the foam deformation. The effective Poisson’s ratio is defined by the axial and lateral strains and can be written as

(7) $$\nu_{\mathrm{eff}}=-\frac{\varepsilon_{L}}{\varepsilon_{U}}=-\frac{\lambda_{L}-1}{\lambda_{U}-1}$$

**Figure 8:**
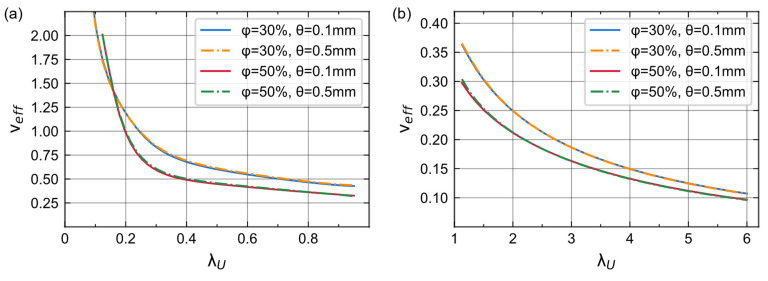
Effective Poisson’s ratio derived from pre-ANN. (**a**) Compression. (**b**) Tension.

The effective Poisson’s ratio of different porosities and cell sizes can be derived from two stretches (Figure 8). Foams with high porosity (φ = 50%) have lower $\nu_{\mathrm{eff}}$ because their λ_L_ changes less with the same λ_U_. Similar to Figure 4b, there is no apparent relationship between the $\nu_{\mathrm{eff}}$ and the cell size. During compression, $\nu_{\mathrm{eff}}$ increases slowly at the beginning but rapidly when λ_U_ reaches a certain threshold. This is because the cells are compressed at the beginning stage of compression, so the increase of lateral stretch is very tiny. After foam densification, the incompressible matrix material makes the lateral stretch increase significantly. During tensile deformation, the effective Poisson’s ratio continuously decreases until it reaches around zero.

**Figure 9:**
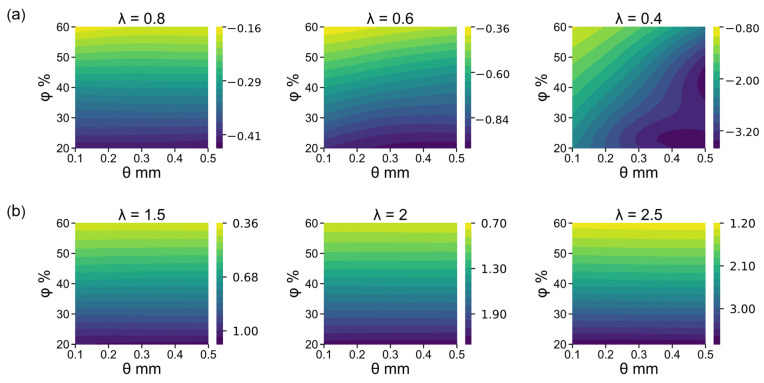
Stress contours of different deformation states. (**a**) Compression. (**b**) Tension.

To investigate the influence of porosity and cell size on the foam’s deformation, we used con-ANN to explore the entire training space. The stress contours of different deformation states are shown in Figure 9. For each axial stretch, the stresses corresponding to 400 different structures can be quickly given by con-ANN. The first row of Figure 9a shows how initial porosity and cell size affect the Cauchy stress during compression. At the start of deformation, stress is only dependent on porosity, but as deformation increases, the impact of cell size on stress becomes increasingly significant. The contour lines are inclined at a certain angle when λ_U_ = 0.6, and the angle becomes more considerable as λ_U_ equals 0.4. Meanwhile, the horizontal contour lines in the second row (Figure 9b) indicate that tensile stress is almost independent of cell size.

## 4. Conclusions

In this work, we proposed an artificial neural network (ANN) constitutive model to describe the mechanical behavior of random elastomeric foams under uniaxial loading, considering the effects of porosity and cell size. Uniform spherical cells were used to represent monodisperse foam. The constitutive relationship between Cauchy stress and the four input variables of axial stretch, lateral stretch, porosity and cell size was given by ANN. After determining the bounds of the input variables, Geodict modeled the foams with different initial porosities and cell sizes and simulated their mechanical response under compression and tension. A number of 100,000 input-output pairs were stored and trained by two ANNs. Lateral stretch was predicted by pre-ANN through the three other variables, and then con-ANN used it as input to predict Cauchy stress. Therefore, researchers could obtain the mechanical responses of their target foam structures using only three input variables. To enable ANN to provide reasonable and interpretable predictions for λ_U_ outside the dataset, we used 1/λ_U_ as the input of pre-ANN and softplus as the activation function of two ANNs.

The results showed that two ANNs perform well on the test data. We can obtain accurate and smooth lateral stretch and axial stress predictions by combining the two ANNs. Pre-ANN can predict the inverse relationship between the two stretches; con-ANN can reasonably explain the λ_U_ outside the dataset. Both ANNs showed some physical interpretability. We employed ANNs to investigate the influence of input variables on lateral stretch and stress: the porosity affects both λ_U_ and stress, while the cell size does not contribute to the value of λ_L_ and is almost irrelevant to the foam tensile response. Compared with polynomial regression and GP regression, con-ANN is proved to have both accurate prediction and interpretable extrapolation. Next, the effective Poisson’s ratio and stress variation trend of different foams under uniaxial loading were given. Finally, although our ANN constitutive model can describe the uniaxial mechanical behavior of random elastomeric foams, there are still several limitations. Experimental data should be added to the dataset to improve the reliability of ANN. The addition of different cell shapes and cell size distributions can broaden the application of the model.

## Figures and Tables

**Table 1:** Range of the input variables.

Input Variables	θ (mm)	φ (%)	λ_U_
Lower bound	0.1	20	0.4
Upper bound	0.5	60	3

**Table 2:** Evaluation metrics of each model on the test data.

Model/Metrics	R^2^	MAPE
pre-ANN	0.999	0.1%
con-ANN	0.997	3.2%
Poly-*n* = 2	0.988	25.9%
Poly-*n* = 3	0.995	13.4%
Poly-*n* = 4	0.9965	6.8%
Poly-*n* = 5	0.9968	3.8%
GPR	0.998	4.2%

## Data Availability

The data presented in this study are available on request from the corresponding author. The data are not publicly available due to they are also part of an ongoing study.
